# Analytical scaling relations to evaluate leakage and intrusion in intermittent water supply systems

**DOI:** 10.1371/journal.pone.0196887

**Published:** 2018-05-18

**Authors:** David D. J. Taylor, Alexander H. Slocum, Andrew J. Whittle

**Affiliations:** 1 Department of Mechanical Engineering, Massachusetts Institute of Technology, Cambridge, MA, United States of America; 2 Department of Civil and Environmental Engineering, Massachusetts Institute of Technology, Cambridge, MA, United States of America; Purdue University, UNITED STATES

## Abstract

Intermittent water supplies (IWS) deliver piped water to one billion people; this water is often microbially contaminated. Contaminants that accumulate while IWS are depressurized are flushed into customers’ homes when these systems become pressurized. In addition, during the steady-state phase of IWS, contaminants from higher-pressure sources (e.g., sewers) may continue to intrude where pipe pressure is low. To guide the operation and improvement of IWS, this paper proposes an analytic model relating supply pressure, supply duration, leakage, and the volume of intruded, potentially-contaminated, fluids present during flushing and steady-state. The proposed model suggests that increasing the supply duration may improve water quality during the flushing phase, but decrease the subsequent steady-state water quality. As such, regulators and academics should take more care in reporting if water quality samples are taken during flushing or steady-state operational conditions. Pipe leakage increases with increased supply pressure and/or duration. We propose using an equivalent orifice area (EOA) to quantify pipe quality. This provides a more stable metric for regulators and utilities tracking pipe repairs. Finally, we show that the volume of intruded fluid decreases in proportion to reductions in EOA. The proposed relationships are applied to self-reported performance indicators for IWS serving 108 million people described in the IBNET database and in the Benchmarking and Data Book of Water Utilities in India. This application shows that current high-pressure, continuous water supply targets will require extensive EOA reductions. For example, in order to achieve national targets, utilities in India will need to reduce their EOA by a median of at least 90%.

## Introduction

Nearly one billion people receive water from piped networks that are not always pressurized [1, 2], such networks are referred to as intermittent water supplies (IWS) and can be contrasted with Continuous Water Supplies (CWS; 24x7 systems) that are standard in most higher-income countries. Since their introduction in the 19th century, the health benefits of CWS have been well recognized [3]. Studies continue to demonstrate that IWS deliver water less equitably and are more likely to recontaminate the finished water than CWS [4, 5]. Yet 41% of piped water systems in lower and middle-income countries operate intermittently [2]. Currently available data show that 97% of utilities in South Asia operate intermittently [6]. In India alone, it is estimated that at least 200 million people drink from IWS [7]. Globally, IWS are responsible for an estimated 17 million infections, 4 million cases of diarrhea, and 1560 deaths annually [1]. Given the prevalence, persistence, and public health burdens of IWS, there is an urgent need to determine operational strategies which maximize water quality in IWS [7, 8].

The operations of IWS can be sub-divided into a *supply period*, when water is being delivered to customers; and a *non-supply period*, when water is left to stagnate and possibly drain out of the pipes through leakage. During the non-supply period, the lack of water pressure allows for contaminant transport into the pipe network through cracks or holes in the pipes [9, 10]. The supply period can be further divided into an initial flushing phase and a steady-state operation phase. Flushing occurs when the water supply is connected, leading to rapid filling of the distribution network and pressurization of pipes. Fluid velocities can be high, causing detachment of biofilms and transport of other accumulated contaminants [7]. Towards the end of the flushing phase, turbidity and contamination decrease over time and eventually reach a steady-state level [7]. While the concentration of fecal indicator bacteria can be 18-20 times higher during the flushing phase, the steady-state phase lasts an average of nine times longer [9]. Therefore, customers’ total contaminant exposure can only be minimized when we understand how operational strategies affect water quality during both phases of supply.

Previous investigations of operational strategies to improve the water quality of IWS have focused on improving the residual chlorine concentration during the steady-state phase [11–13] and identifying likely locations of contaminant intrusion during the non-supply period [10, 14]. None of these approaches explore how operational changes would affect water quality during both the flushing and steady-state phases of the system.

First-order models, despite their many simplifications, can provide important insights into complex systems [15]. Accordingly, we propose a simplified model that can be used to investigate strategies to improve water quality in IWS, through equations that couple supply duration, supply pressure, and leakage rate. We apply the model to determine the required extent of leak repair as supply duration and/or pressure is improved under water-scarce scenarios. *Second*, we extend these scaling equations to study how supply duration, supply pressure, and leak repair can affect the volume of intruded, potentially-contaminated fluids present during the flushing and steady-state phases. And *third*, we quantify the implications of these scaling equations by applying them to self-reported performance indicators for IWS serving 108 million people.

## Simplifying assumptions and first-order models

### Background

Galaitsi et al. [16] classify three types of IWS: *predictable*, where customers receive a predictable volume of water according to a known schedule; *irregular*, where the received volume is predictable, but the schedule is unknown; and *unreliable*, where the received volume and schedule are both uncertain. Customers who have access to enough water in predictable IWS, typically either wait until flushing water has traveled downstream, or discard the flushing water. Unfortunately, disadvantaged customers in predictable IWS and customers in irregular or unreliable IWS cannot trust the system enough to discard the flushing water; this leaves them exposed to increased levels of contamination. Additionally, some customers collect water directly in underground or rooftop tanks (which are promoted by irregular and unreliable IWS) and hence capture the flushing water and its contaminants [7].

Elala et al. [17] and Kumpel and Nelson [9] suggest four modes by which IWS can degrade water quality: *contaminant intrusion*, where contaminants enter the system from the vicinity of the distribution pipes; *biofilm growth and/or sloughing*, where the hydraulic conditions of IWS encourage biofilm growth and/or detachment; *domestic storage*, where IWS force customers to use domestic storage containers which create opportunities for recontamination through poor hygiene; and *backflow*, where contaminants enter into the system from customer premises. Kumpel and Nelson [5, 9] have confirmed that intrusion is a likely contamination mechanism. Accordingly, this paper considers the factors which affect the volume of contaminants that can intrude into IWS.

Contaminants can only intrude into a pipe network when: i) there are physical pathways (at leaky joints, or through fractures in the pipes), ii) there are contaminants in the same vicinity, and iii) there is an inward pressure gradient [18]. The risk of contaminant intrusion in CWS is typically calculated using: i) the outward leakage rate to assess the size of physical pathways; ii) the assumption of ubiquitous contaminants; and iii) the measured or modeled magnitude and duration of low pressure events [19–21]. This approach conservatively estimates the maximum volume of fluid that could intrude into the water supply system without specifically considering the concentration (if any at all) of contaminants in the intruding fluid. We extend this standard approach by explicitly considering the duration of supply and by distinguishing between the flushing and steady-state phases.

### Leaks and intrusion

Wherever a pathway exists between the interior and exterior of a pipe, the potential for outward leakage and inward intrusion exist. For a given pathway, both inward and outward flows cannot occur simultaneously. However, across a variably-pressurized network surrounded by externally-pressurized fluids, inward and outward flows may occur simultaneously at different locations. To simplify the model that follows, we account for these flows independently, which ensures that the equations describing each are always greater than or equal to zero.

We model a system’s volume of leakage (*V*_*L*_) using a power law equation which depends on the average system pressure head (*H*) and the duration of the supply period (*t*, in hours):

$$\begin{array}{c} V_{L}=tQ_{L}=tk_{L}AH^{\alpha } \end{array}$$ (1)

Where *A* is the equivalent orifice area (EOA) that a single orifice would have if its leakage rate matched the system’s leakage rate (*Q*_*L*_), *k*_*L*_ is a combined constant, and *α* accounts for the pressure dependency of the flow rate. We take *α* = 1.0 as is standard practice [22]. Implicitly, Eq 1 assumes that the outward leakage rate is not significantly affected by the pressure of external fluids.

By a similar approach, the volume of potentially-contaminated fluid that intrudes during the non-supply period (i.e., when *H* = 0), *V*_*CF*_, is:

$$\begin{array}{c} V_{CF}=\left(24-t\right)Q_{CF}=\left(24-t\right)k_{C}f_{C}AH_{C}^{\beta } \end{array}$$ (2)

Where *Q*_*CF*_ is the intrusion rate during the non-supply period, *f*_*C*_ is the probability that external fluids are in the vicinity of a given leakage/intrusion pathway, *k*_*C*_ is a combined constant, and *H*_*C*_ is the average external pressure of fluids surrounding the pipe (e.g., groundwater pressure). *β* accounts for the pressure dependency of the rate of intrusion.

Accounting for the intrusion potential during the supply period is more complex. In an IWS, pressure varies substantially throughout the network and so even when the average system pressure head (*H*) is higher than the average pressure of fluids surrounding the pipe (*H*_*C*_), some intrusion may still occur (in locations where the internal pipe pressure is less than the external fluid pressure). In order to account for this possibility, we model the network pressure at a given leakage/intrusion pathway with an unknown probability distribution *f*(). The intrusion rate during the supply period, *Q*_*C*_, is therefore:

$$\begin{array}{c} Q_{C}=k_{C}f_{C}A\int_{-\infty }^{\infty }f\left(H\right)\text{min}\left(0,{\left(H_{C}-H\right)}^{\beta }\right)dH \end{array}$$ (3)

To simplify the algebra that follows, we represent this probabilistic model with the function *ϕ*(). The volume (of potentially-contaminated fluid) which intrudes during the supply period (*V*_*C*_) is, therefore:

$$\begin{array}{c} V_{C}=tQ_{C}=tf_{C}A\phi \left(H_{C}-H\right) \end{array}$$ (4)

More detailed derivations of Eqs 1, 2 & 3 can be found in S1 Text.

*Leak repair strategy:* We assume that when utilities reduce their EOA, they do so without strategically considering the location or pressure of potential intrusion sources. This assumption allows the ratio of leak pathways with intrusion sources in their vicinity (*f*_*C*_) to remain constant during system improvements. Unfortunately, this also prevents our model from capturing the importance of removing high-risk intrusion sources (e.g., eliminating cross-connections between water pipes and sewers) [10].

### Intruded volume and the fate of intruded contaminants

We model factors which govern the total volume of fluids intruding into the system, but do not differentiate between the concentration or health risks of intrusion sources. Similarly, we do not account for the possibility that intrusion sources could be depleted through intrusion or diluted through leakage. This approach is consistent with risk assessments for CWS [19–21, 23].

Since we consider only the intruded volume, we implicitly treat any contaminants it contains as conserved species, neglecting disinfection and biomass growth. Similarly, we also neglect the potential for the storage of contaminants in the biofilm, which may store pathogens and release them at a later time [24] (e.g., sloughing of biofilms during flushing and steady-state phases).

While we model intrusion during both supply and non-supply periods, we assume that all of the intruded volume (and all of its contaminants) exit the system exclusively through customer premises (i.e., no intruded volume leaks out of the system). This implies that the intruded volume which accumulated during non-supply (*V*_*CF*_) is also the intruded volume present during the flushing phase.

*Limitations:* Neglecting biofilm growth, storage, and sloughing may underestimate the effect that fluids which intrude during non-supply have on steady-state water quality. Conversely, neglecting disinfection may overestimate the importance of reducing intruded fluids in systems with consistently-high concentrations of residual disinfectant. Finally, neglecting the quality and potential dilution of intrusion sources may overestimate the importance of minimizing the potential flux of intruded fluids.

*Quantification:* We use log reduction (LR) to account for the relative reduction in the volume of intruded (and potentially-contaminated) fluids from an original volume, $V_{C}^{0}$, to the final, $V_{C}^{*}$ [25]:

$$\begin{array}{c} \text{LR}=\log_{10}\left(\frac{V_{C}^{0}}{V_{C}^{*}}\right)=-\log_{10}\left(\frac{V_{C}^{*}}{V_{C}^{0}}\right) \end{array}$$ (5)

A negative LR value suggests that intrusion (and its risk of contamination) has increased. For additional details on LR, see S1 Text. Implicitly, this metric assumes that $V_{C}^{0}$ is strictly positive (i.e., $V_{C}^{0}>0$).

### Flushing phase’s instantaneous, unmixed flow

Intrusion can occur during the non-supply period, the flushing phase, and the steady-state phase of an IWS. However, in light of the limited duration of the flushing phase [9], our model assumes that the flushing phase is instantaneous (i.e., the steady-state phase lasts for *t*). In addition, despite the continuous transition between flushing and steady-state phases, we model these two phases as distinct and do not account for mixing of the flushing phase with the steady-state phase.

The first assumption (instantaneous flushing) overestimates the potential volume of intruded fluids in the steady-state phase and underestimates it in the flushing phase. The second assumption (no mixing) has the opposite effect.

### Simplifying an IWS to an equivalent node

As a departure point for more detailed models, we model an IWS as a single supply pipe with all of its customer demand concentrated at its end. Blokker et al. [26] showed that aggregating several hundred customers’ demand into bulk nodes in CWS simulations had little effect on the simulated flow and dispersion, for timescales longer than 30 minutes. We extend far beyond their scope of aggregation, following Abu-Madi and Trifunovic [27], and aggregate all customers into a single demand at the end of a single supply pipe.

Along a single pipe, Colombo and Karney [28] showed that the leakage location, end-point pressure, and end-point demand all affect the leakage rate. These interactions simplify greatly, however, if we assume either that: leaks are located at the end of the pipe, or the flow is inviscid [28].

Due to the high number of joints, the variability of construction materials, and lack of inspection, “worldwide, the majority of leakage events and… volume losses occur at the customer service connection” [22]. Therefore, we further simplify our model by assuming that all leakage occurs at the end of the single pipe. This overestimates the increase in leakage that occurs if customer pressure is increased [28].

**Fig 1:**
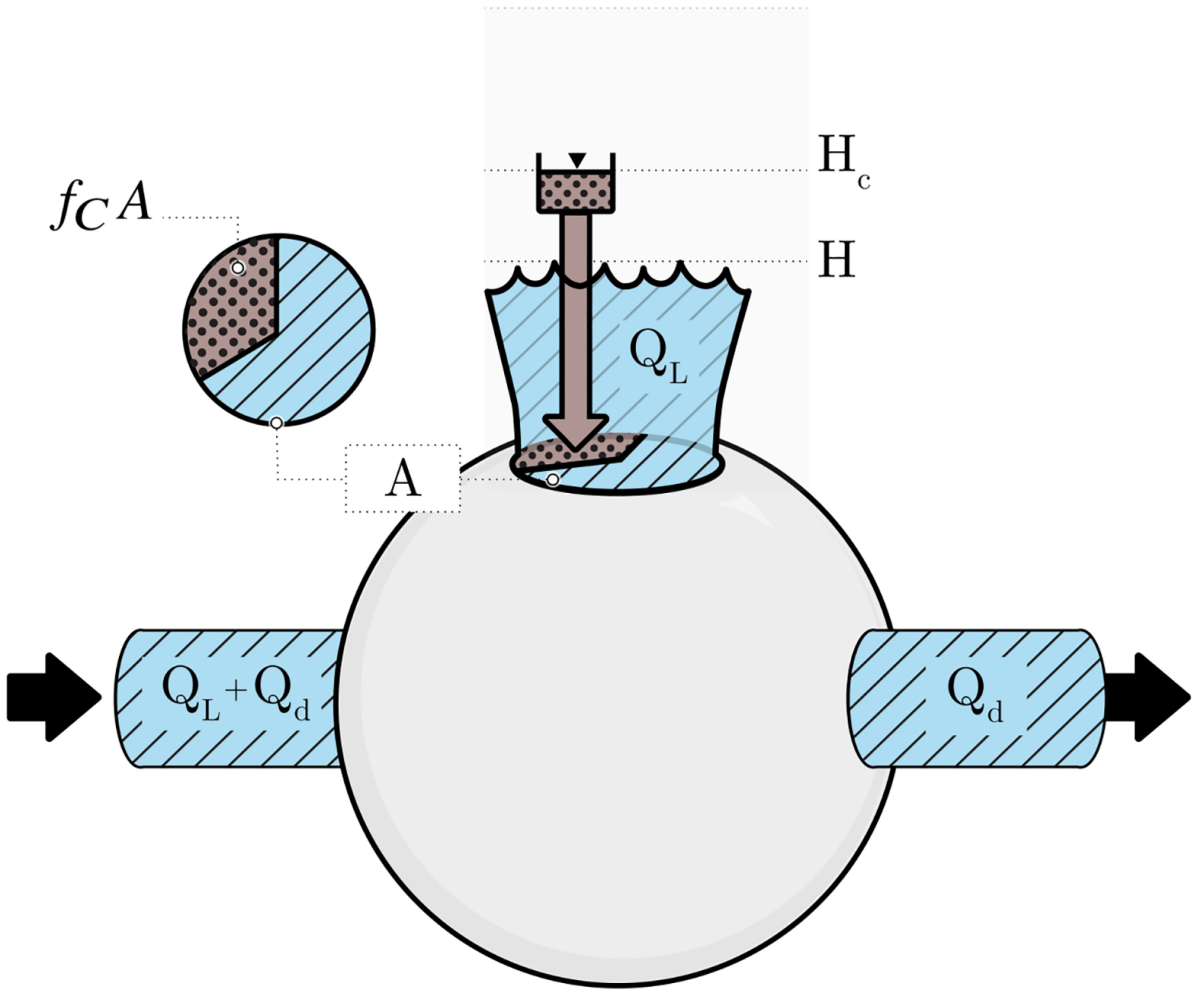
Single node equivalent of an IWS. We model the system or sub-system as an equivalent node, with pressure head *H*, average external fluid pressure *H*_*C*_, flow to customers *Q*_*d*_, and leakage flow *Q*_*L*_; *A* is the equivalent area of pathways for leakage and *f*_*C*_*A* is the equivalent area for intrusion.

With all demand and leakage pathways concentrated at the end of the pipe, our first-order model reduces to an equivalent node, with a single pressure, demand, area for leakage, prevalence of intrusion sources, and average external fluid pressure, as shown in Fig 1.

### Allowable increases in leakage

We introduce the variable *l* to account for a utility’s ability to accommodate additional leakage in their system, from its initial value ($V_{L}^{0}$) to its final ($V_{L}^{*}$), as a percentage of the initial volume of water input into the system ($V_{T}^{0}$):

$$\begin{array}{c} l\geq \frac{V_{L}^{*}-V_{L}^{0}}{V_{T}^{0}}=\frac{V_{L}^{0}}{V_{T}^{0}}(\frac{V_{L}^{*}}{V_{L}^{0}}-1) \end{array}$$ (6)

Most IWS are constrained by the volume of water they have available and cannot allow leakage to increase (i.e., *l* = 0). However, *l* can be positive if system improvements lead to increases in finished water production (*V*_*T*_) or diversions of water from other areas, or if the total water consumption (*V*_*d*_) (paying plus non-paying customers) decreases. Conversely, if consumption increases, *l* can become negative.

### Leakage metrics: NRW, UFW, and EOA

Non-revenue water (NRW) is the amount of water that is distributed, but does not generate revenue; it is often used as a proxy for leakage rates. Unaccounted for water (UFW) tracks how much water is missing from a utility’s water balance of input minus output. In order to make use of the available data, we neglect the difference between UFW and NRW, which is usually small [6].

Despite the limitations of accounting for NRW (*N*) as a percentage of total input volume [29], we continue to do so in order to use the reported NRW values from the available databases. To apply the proposed equations, an assumption is required about what fraction (*p*) of each utility’s NRW is due to physical leakage. The fraction of the input volume which is lost to (physical) leakage is:

$$\begin{array}{cc} V_{L}=pNV_{T} & \left(0\leq p\leq 1\right) \end{array}$$ (7)

### Data sources

We combine self-reported water utility data from the World Bank’s IBNET database [6] and the 2007 Benchmarking and Data Book of Water Utilities in India (BDBWUI) [30]. Filtering for outliers and excluding missing data, as described in S1 Text, we arrive at Table 1. These datasets represent 334 utilities, serving 108 million people with IWS. Unfortunately, the IBNET database does not include a metric for pressure. We will highlight four cities, each near the 25th or 75th percentile of their datasets (Table 2).

**Table 1:** Characteristics of IWS in the filtered IBNET and BDBWUI databases, subtotaled and combined.

Region	Countries n	Utilities n	Population Served mean (range) total ×10^6^	Supply Duration mean (range)	NRW mean (range)	Pressure (m) mean (range)	Year (range)	Database
EAP^a^	3	22	0.51 (0.031-7.1) 11.1	18.4 (2.0-23.0)	32% (19-62%)	N.R.^b^	(2009-15)	IBNET
ECA^a^	8	76	0.06 (0.002-0.8) 4.4	13.7 (3.0-23.5)	47% (4-80%)	N.R.^b^	(2001-14)	IBNET
LAC^a^	3	20	0.60 (0.022-7.5) 12.0	16.6 (4.0-23.0)	50% (34-69%)	N.R.^b^	(2006-13)	IBNET
MENA^a^	3	11	0.42 (0.018-2.6) 4.6	10.4 (3.0-20.0)	29% (18-40%)	N.R.^b^	(2010)	IBNET
SA^a^	1	46	0.12 (0.003-1.4) 5.4	8.0 (2.0-16.0)	19% (3-45%)	N.R.^b^	(2014)	IBNET
SSA^a^	11	150	0.38 (0.004-4.8) 57.3	13.0 (1.4-23.5)	34% (4-79%)	N.R.^b^	(2011-14)	IBNET
IBNET total	29	325	0.29 (0.002-7.5) 94.9	13.0 (1.4-23.5)	36% (3-80%)	N.R.^b^	(2001-15)	IBNET
BDBWUI total	India	9	3.8 (0.6-13.0) 13.0	5.5 (1.0-11.0)	30% (13-57%)	4.3 (2.0-10.0)	(2005-06)	BDBWUI
Total	30	334	0.38 (0.002-7.5) 107.9	12.8 (1.0-23.5)	36% (3-80%)	N.A.^c^	(2001-15)	*Both*

^a^ East Asia and the Pacific (EAP), Europe and Central Asia (ECA), Latin America and the Caribbean (LAC), Middle East and North Africa (MENA), South Asia (SA), Sub-Saharan Africa (SSA).

^b^ Not reported

^c^ Not applicable

**Table 2:** Summary data for case study cities.

Country	City	Population ×10^6^	Continuity (hrs/day)	NRW	Pressure (m)	Reporting Year	Dataset
Tanzania	Dar es Salaam	1.93	8	56%	N.R.^a^	2013	IBNET
Yemen, Rep.	Hajjah	0.05	18	24%	N.R.^a^	2010	IBNET
India	Mumbai	13.00	4	13.6%	7	2005-06	BDBWUI
India	Varanasi	1.60	7	30%	3	2005-06	BDBWUI

^a^ Not reported

## Scaling relations for pipe networks

### Required reductions in EOA

Substituting Eqs 1 and 7 into Eq 6:

$$\begin{array}{lll} pN\left(\frac{t^{*}}{t^{0}}{\left(\frac{H^{*}}{H^{0}}\right)}^{\alpha }\frac{A^{*}}{A^{0}}-1\right) & \leq & l\\ ∴\frac{A^{*}}{A^{0}} & \leq & \left(\frac{t^{0}}{t^{*}}\right){\left(\frac{H^{0}}{H^{*}}\right)}^{\alpha }\left(\frac{l}{pN}+1\right) \end{array}$$ (8)

Each of the three terms in parentheses in Eq 8 refers to the scaling of EOA necessitated by a different aspect of system improvement: supply duration, supply pressure, and additional leakage allowance. Increased supply pressure and/or duration increases the necessitated repairs, while an additional leakage allowance (e.g., because of extra water available from a water treatment plant expansion) reduces them.

As proposed, the repair requirement suggests that utilities can allow their EOA to increase (e.g., by ceasing leak repair) if they i) also allow leakage to increase (e.g., by supplying extra water), ii) reduce supply duration, and/or iii) reduce supply pressure (Eq 8). This may explain why low-pressure IWS are prevalent as they provide an easy alternative to the major infrastructure improvements needed to reduce EOA.

In this paper we focus on the system improvement process and therefore assume that EOA does not increase (i.e., $\frac{A^{*}}{A^{0}}\leq 1$):

$$\begin{array}{c} ∴\frac{A^{*}}{A^{0}}\leq \text{min}\left[1,\frac{t^{0}}{t^{*}}{\left(\frac{H^{0}}{H^{*}}\right)}^{\alpha }\left(\frac{l}{pN}+1\right)\right] \end{array}$$ (9)

Assuming that utilities do as little work as possible (i.e., maximizing A* subject to Eq 9):

$$\begin{array}{c} \frac{A^{*}}{A^{0}}=\text{min}\left[1,\frac{t^{0}}{t^{*}}{\left(\frac{H^{0}}{H^{*}}\right)}^{\alpha }\left(\frac{l}{pN}+1\right)\right] \end{array}$$ (10)

In practice, Eq 10 is not universally applicable. For example, the CWS pilot project in Hubli-Dharwad replaced the entire pipe network (A* ≈ 0) [31]. Nevertheless, we move forward with this assumption as most capital improvement projects do not involve complete replacement of the pipe network.

### Effect of reduced EOA

Since the EOA for leaks (*A*) is common to Eqs 1, 2 and 4, these equations suggest that reducing *A* by the fraction $\frac{A^{*}}{A^{0}}$ (in the case where t* = *t*^0^ and H* = *H*^0^) may also scale the volume of intruded fluids in the steady-state and flushing phases by the ratio $\frac{A^{*}}{A^{0}}$, provided the initial volume of each is non-zero (see S2 Text for derivation):

$$\begin{array}{ccc} \frac{V_{C}^{*}}{V_{C}^{0}}{|}_{t^{*}=t^{0},H^{*}=H^{0}}=\frac{V_{CF}^{*}}{V_{CF}^{0}}{|}_{t^{*}=t^{0}}=\frac{V_{L}^{*}}{V_{L}^{0}}{|}_{t^{*}=t^{0},H^{*}=H^{0}}=\frac{A^{*}}{A^{0}} & & :V_{C}^{0},V_{CF}^{0},V_{L}^{0}>0 \end{array}$$ (11)

### Effect of increased supply duration

*Steady-state:* Eq 4 suggests that an increase in the supply period duration from *t*^0^ → t* (with other parameters held constant) may increase the volume of contaminants intruding into the system during steady-state by $\frac{t^{*}}{t^{0}}$, provided $V_{C}^{0}>0$:

$$\begin{array}{ccc} \frac{V_{C}^{*}}{V_{C}^{0}}{|}_{A^{*}=A^{0},H^{*}=H^{0}}=\frac{t^{*}f_{C}A^{0}\phi \left(H_{C}-H^{0}\right)}{t^{0}f_{C}A^{0}\phi \left(H_{C}-H^{0}\right)}=\frac{t^{*}}{t^{0}}>1 & & :V_{C}^{0}>0 \end{array}$$ (12)

**Fig 2:**
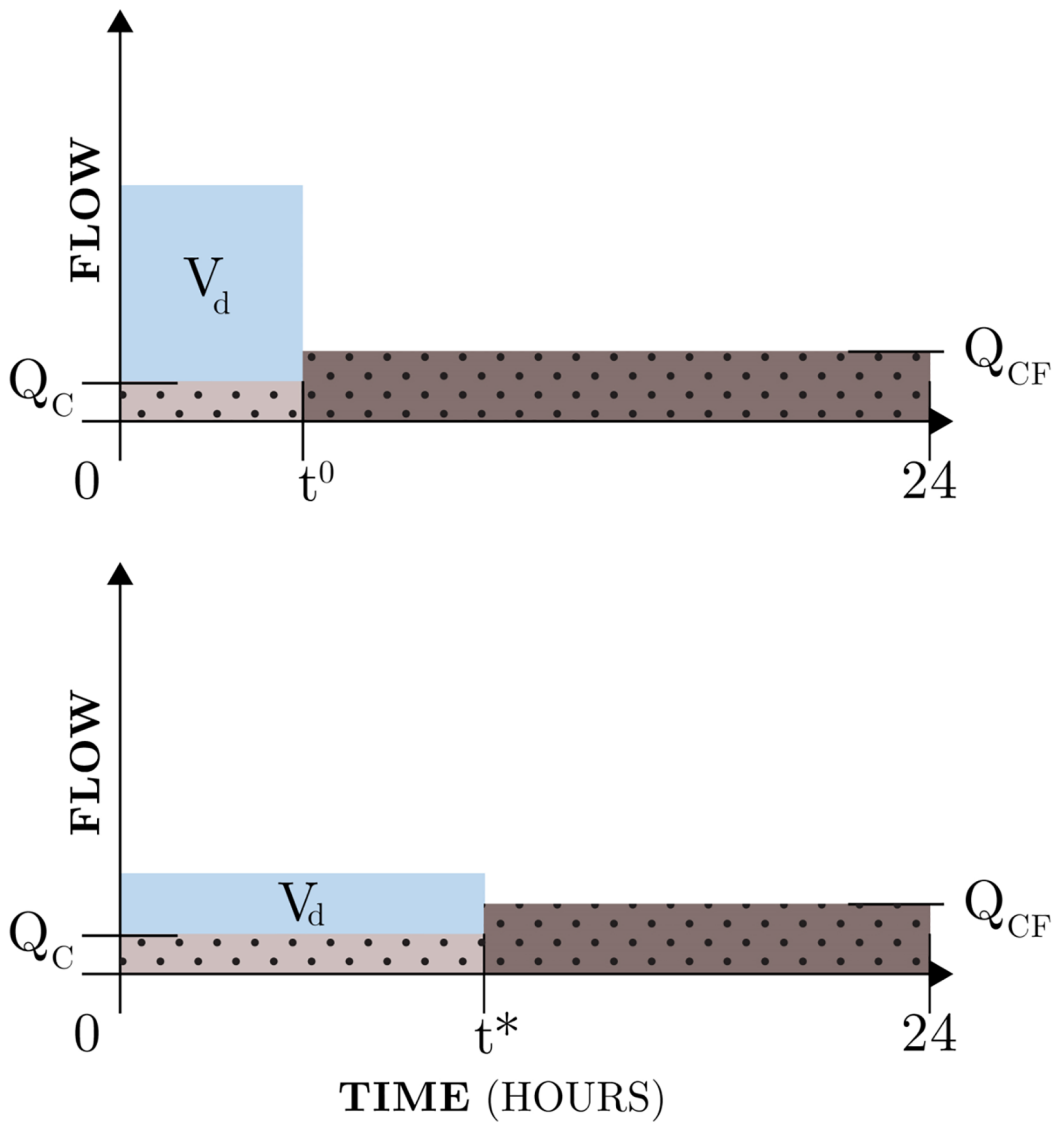
Supply duration’s effect on intruded fluids. Compare an initial system (top panel) with supply period duration *t*^0^, customer demand volume *V*_*d*_, and contaminant intrusion rates *Q*_*C*_ and *Q*_*CF*_ during the supply and non-supply periods, to the same system (bottom panel) if the supply period duration is lengthened (to t*). If all-else is held constant, *Q*_*C*_ and *Q*_*CF*_ are also constant.

To our knowledge, this relationship has not been noted in the literature on IWS, and contradicts the conventional belief that increasing the supply period duration is universally good for water quality. If all other factors are held constant, the intrusion rate into the system during the supply period is independent of the supply period duration (*t*); the accumulated volume is therefore linearly dependent on the supply period duration (Eq 12), as highlighted in Fig 2. For systems where customer demand does not significantly depend on the duration of supply (e.g., customer demand may vary by only 15-20% where supply durations vary between 6 and 24 hrs/day [32, 33]), Eq 12 additionally suggests that the concentration of intruded fluid in the steady-state phase may also increase with the supply period duration. We note that Eq 12 does not suggest that water quality will degrade by converting a low-pressure IWS to a high-pressure CWS, but instead suggests that in two systems with equal pressure distributions, differing only in supply duration, the one with the longer duration will allow more time for any steady-state intrusion to accumulate.

Our simplifying assumptions obscure two mechanisms which could temper this finding: first, we neglect contaminant storage and growth by which intrusion during the non-supply period could influence steady-state water quality. Second, we fix pressure at the location of intrusion; otherwise, increased supply duration (which reduces flow rates and therefore friction losses) would increase pressure and therefore reduce the intrusion rate during the steady-state phase. Adding these mechanisms could provide useful refinements of the current model.

*Flushing:* In keeping with conventional understanding, Eq 2 suggests that increasing the supply duration may decrease the volume of fluids that intrudes during the non-supply period and which is therefore present during the flushing phase by $\frac{24-t^{*}}{24-t^{0}}<1$ (e.g., Fig 2).

*Steady-state and flushing combined:* Some customers are impacted by the total volume of intruded fluids in the system during steady-state and flushing phases. Assuming t* > *t*^0^ and $(V_{C}^{0}+V_{CF}^{0})>0$, the combined intruded volume scales as:

$$\begin{array}{ccc} \frac{V_{C}^{*}+V_{CF}^{*}}{V_{C}^{0}+V_{CF}^{0}}{|}_{A^{*}=A^{0},H^{*}=H^{0}}=\frac{t^{*}(\frac{Q_{C}}{Q_{CF}}-1)+24}{t^{0}(\frac{Q_{C}}{Q_{CF}}-1)+24}\underset{\left(\text{if}\;Q_{CF}\geq Q_{C}\right)}{\leq 1} & & :(V_{C}^{0}+V_{CF}^{0})>0 \end{array}$$ (13)

If *H*^0^ ≥ 0 everywhere in the system ($\int_{-\infty }^{0}f\left(H\right)dH=0$), the condition *Q*_*CF*_ ≥ *Q*_*C*_ is always met; more generally, *Q*_*CF*_ ≥ *Q*_*C*_ whenever Eq S7 (from S2 Text) holds. This qualification (*H*^0^ ≥ 0) is important as some neighborhoods have sub-atmospheric supply pressures due to customers using suction pumps [7, 34].

*Impact:* Volumes of flushed water (and any associated contaminants) are not evenly distributed between customers. If supply durations are increased, customers that do not currently consume any flushing water will have a higher risk of contaminant exposure. Conversely, customers that currently consume substantial volumes of flushing water will have a reduced risk of contaminant exposure.

Distinguishing between these effects has not been discussed in the literature and may help understand the health impacts of CWS: Ecrumen et al. [35] found that CWS only had significant health benefits for lower-income families, which they hypothesized might be due to more frequent usage of water filters in higher-income families. Our model suggests another plausible mechanism: if lower-income families were exposed to flushing water more than higher-income families, the water quality improvements due to CWS would be concentrated in lower-income families.

Similarly, while Adane et al. [36] found a strong association (adjusted odds ratio of 4.8) between IWS and acute diarrrhea in children under five in slums in Addis Ababa, they found 94% of the water quality samples (across IWS and CWS) to be of low risk for *E. coli* contamination. While household storage is known to reduce water quality [7], the IWS-induced risk of diarrhea could also have been caused by lower quality flushing water, missed by their sampling strategy, which did not distinguish between flushing and steady-state phases.

More generally, for utilities that only sample water quality in steady-state conditions (a common practice), the scaling equations suggests that the measured water quality would worsen after converting to CWS (assuming no increase in pressure or reduction in EOA). Regulations, utilities, and researchers studying IWS should specify more carefully which phase of supply is to be, or has been, measured (e.g., this has been reported by some authors [5, 37], but not others [8, 36, 38]).

### Combined effects of leak repair and supply duration

*Steady-state:* When increased supply duration is considered in combination with necessary reductions in EOA (to prevent an increase in *V*_*L*_), the net effect on the intruded volume during the steady-state phase depends on allowable increase in leakage (*l*). Combining Eqs 4 and 10, and assuming $V_{C}^{0}>0$:

$$\begin{array}{lll} \;\frac{V_{C}^{*}}{V_{C}^{0}}{|}_{H^{*}=H^{0}} & =\frac{t^{*}f_{C}\phi \left(H_{C}-H^{0}\right)}{t^{0}f_{C}\phi \left(H_{C}-H^{0}\right)}\text{min}\left[1,\frac{t^{0}}{t^{*}}{\left(\frac{H^{0}}{H^{0}}\right)}^{\alpha }\left(\frac{l}{pN}+1\right)\right] & :V_{C}^{0}>0\\ ∴\frac{V_{C}^{*}}{V_{C}^{0}}{|}_{H^{*}=H^{0}} & =\frac{t^{*}}{t^{0}}\text{min}\left[1,\left(\frac{t^{0}}{t^{*}}\right)\left(\frac{l}{pN}+1\right)\right] & :V_{C}^{0}>0 \end{array}$$ (14)

In the limit where a utility cannot allow leakage to increase (i.e., *l* = 0), and must therefore conduct all of the necessary EOA reductions, Eq 14 suggests that increased supply duration will have no net effect on the intruded volume in the steady-state phase ($\frac{t^{*}}{t^{0}}\frac{t^{0}}{t^{*}}=1$). However, if a utility does less EOA reduction (perhaps by expanding its water supply capacity), Eq 14 suggests that increased supply duration will increase the intruded volume in the steady-state phase.

Consider, for example, a utility with enough extra water to allow its leakage to increase by 50% ($\frac{l}{pN}=0.5$). While such an increase may seem unreasonable, it can occur if the utility had 40% NRW, of which 50% was due to physical losses. In this case, a reasonable increase in production capacity of 10% would allow for a 50% leakage increase (i.e., $\frac{0.1}{0.5*0.4}=0.5$). Assuming that some intrusion occurs during both supply and non-supply periods, Eqs 5 and 12 suggest that if the supply duration is increased from *t*^0^ = 6 to t* = 21 hrs/day, the utility would have a log reduction (LR) = $-log_{10}\left(\frac{t^{*}}{t^{0}}\right)=-0.54$ due to increased supply duration alone. However, Eq 14 also suggests that the LR from the combined effects of supply duration and EOA reduction would be $-log_{10}\left(\frac{w}{pN}+1\right)=-0.17$. In both cases, the proposed equations suggest that the intruded volume in the steady-state phase will increase. We plot a range of other scenarios in S1 Fig.

*Flushing:* The net effect of increasing the supply duration and its associated EOA reduction on the intruded volume in the flushing phase is modeled by Eqs 2 and 10:

$$\begin{array}{lll} \;\frac{V_{CF}^{*}}{V_{CF}^{0}}{|}_{H^{*}=H^{0}} & =\frac{\left(24-t^{*}\right)k_{C}f_{C}H_{C}^{\beta }}{\left(24-t^{0}\right)k_{C}f_{C}H_{C}^{\beta }}\text{min}\left[1,\frac{t^{0}}{t^{*}}{\left(\frac{H^{0}}{H^{0}}\right)}^{\alpha }\left(\frac{l}{pN}+1\right)\right] & :V_{CF}^{0}>0\\ ∴\frac{V_{CF}^{*}}{V_{CF}^{0}}{|}_{H^{*}=H^{0}} & =\frac{\left(24-t^{*}\right)}{\left(24-t^{0}\right)}\text{min}\left[1,\frac{t^{0}}{t^{*}}\left(\frac{l}{pN}+1\right)\right] & :V_{CF}^{0}>0 \end{array}$$ (15)

**Fig 3:**
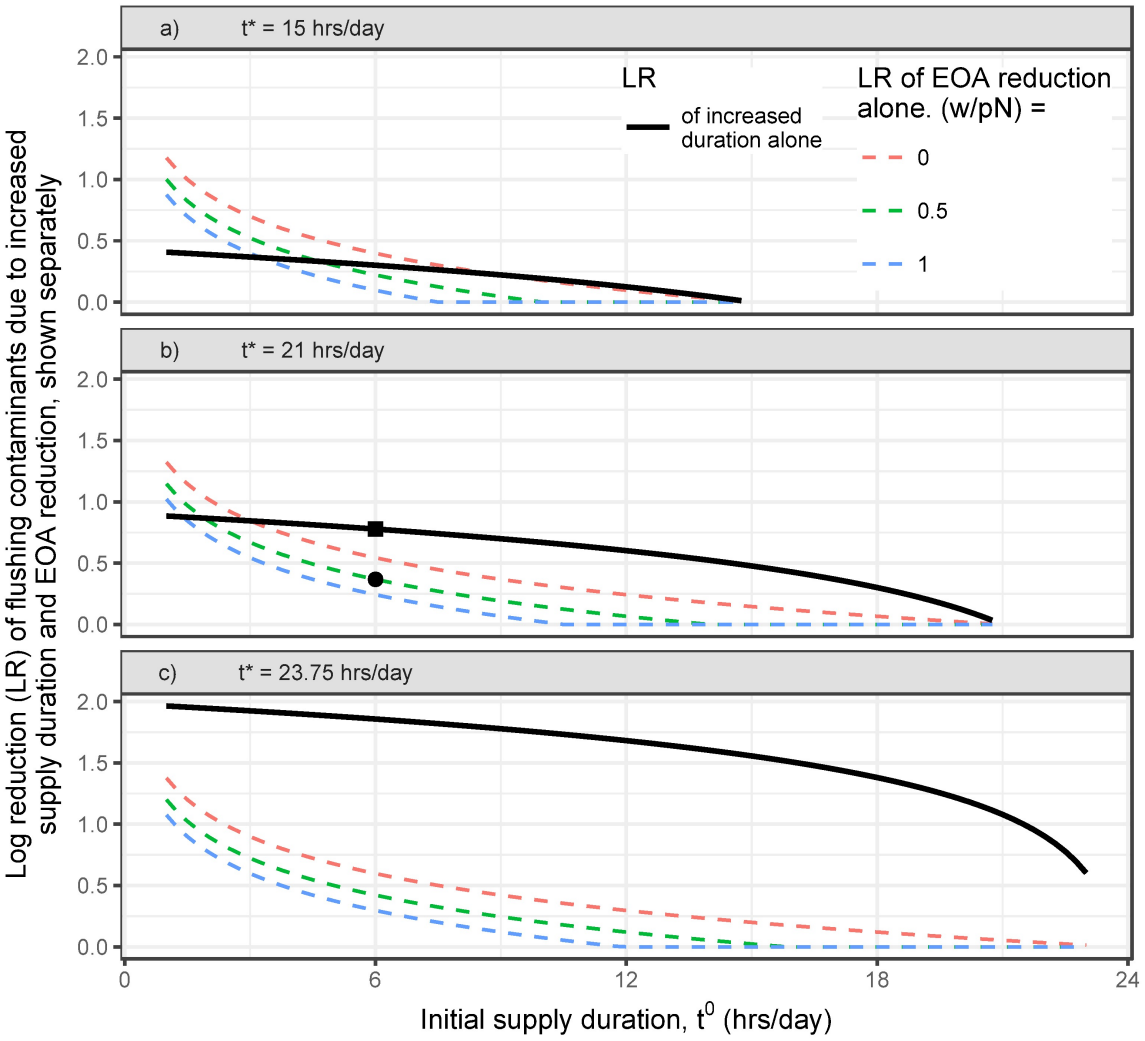
LR during the flushing phase from increased supply duration and reduced EOA, separately. The LR during flushing attributable to increased supply duration (thick black line and square dot), and due to the necessitated reductions in EOA (colored dashed curves), each plotted separately. Three levels of allowable leakage increases are shown: no increase (pink/upper curves), a 50% increase in physical losses (i.e., $\frac{l}{pN}=0.5$) (green/middle curves, and circle), and a 100% increase in physical losses (blue/lower curve). Final durations of 15, 21, and 23.75 hrs/day are shown in the top (a), middle (b), and bottom (c) panels, respectively. The text’s example utility is also shown (square and circle).

The predicted effect from increased supply duration is $\frac{24-t^{*}}{24-t^{0}}$, while EOA reduction’s effect is $\frac{t^{0}}{t^{*}}(\frac{l}{pN}+1)$. Both effects act to reduce intrusion-induced risk in (i.e., improve the safety of) the flushing phase; we therefore compare their relative magnitudes. For the example utility introduced above, Eqs 2 and 10 suggest a LR in the intruded volume in the flushing phase due to increased supply duration of $-log_{10}\left(\frac{24-t^{*}}{24-t^{0}}\right)=0.78$, and a LR from EOA reduction of $-log_{10}\left(\frac{t^{0}}{t^{*}}(\frac{l}{pN}+1)\right)=0.37$. For this example, the increased supply duration is expected to be substantially more important than EOA reduction for improving the safety of the flushing phase. Fig 3 shows these two predicted effects separately for a range of simulated utilities.

As the increased supply duration t* → 24 hrs/day, its effect on the safety of the flushing phase is expected to dominate over the effect of reduced EOA (Eq 15). Indeed, Eq 15 suggests that if t* = 21 hrs/day, supply duration dominates over the importance of reducing EOA for all cases with initial supply durations *t*^0^ ≥ 3 hrs/day (Fig 3b). Conversely, if the final supply duration is brief, the necessary reductions in EOA may also substantially contribute to improved flushing safety: for example, if the final supply duration t* = 15 hrs/day (Fig 3a), reducing EOA becomes more important in systems where leakage cannot increase substantially (i.e., $\frac{l}{pN}\leq 0.5$) and with initial supply durations *t*^0^ ≤ 9 hrs/day (Fig 3a).

*Impact:* Eqs 2 and 10 suggest that utilities which want to improve the safety of their flushing water while operating with t* ≤ 15, should develop campaigns to reduce EOA and then reassess if additional contaminant reduction is required. More generally, our model suggests that projects which increase their supply duration, should reduce their EOA by at least $\frac{t^{0}}{t^{*}}$ to preserve steady-state quality. Carefully monitoring EOA reductions should therefore be a priority for regulators and project managers.

Neither of the two recent cross-sectional studies of water quality in IWS are able to assess the steady-state predictions of the scaling equations. Kumpel and Nelson [5] examined the water quality of a project that replaced 100% of the pipe network [31], rendering A* ≈ 0 (vs. Eq 10). Such a massive reduction in the EOA would offset the predicted harmful effects of increased supply duration on steady-state water safety. Erickson et al. [37] studied IWS in Arraiján, Panama, and did not find significant differences in steady-state water quality between zones with different supply period durations (likely due to high water quality). However they did find deterioration in the water quality of the flushing phase when the duration of the non-supply period increased, as predicted (although the variance in this trend was high).

### Effect of increased pressure

Since we neglect contaminant storage and growth, supply pressure has no effect on the volume of fluids that accumulate during the non-supply period (Eq 2) and therefore system pressure has no direct effect on the intruded volume in the flushing phase (*V*_*CF*_). Conversely, due to the structure of *ϕ*(), a uniform increase in pressure cannot increase steady-state intrusion-induced risk. Assuming $V_{C}^{0}>0$:

$$\begin{array}{ccc} \frac{V_{C}^{*}}{V_{C}^{0}}{|}_{A^{*}=A^{0},t^{*}=t^{0}}=\frac{t^{0}f_{C}A^{0}\phi \left(H_{C}-H^{*}\right)}{t^{0}f_{C}A^{0}\phi \left(H_{C}-H^{0}\right)}=\frac{\phi (H_{C}-H^{*})}{\phi (H_{C}-H^{0})}\leq 1 & & :V_{C}^{0}>0 \end{array}$$ (16)

In practice, this relationship has been observed to have a threshold after which the system pressure exceeds any plausible external fluid pressure and there are no further improvements in water quality [9, 18].

### Summary of the scaling equations

**Fig 4:**
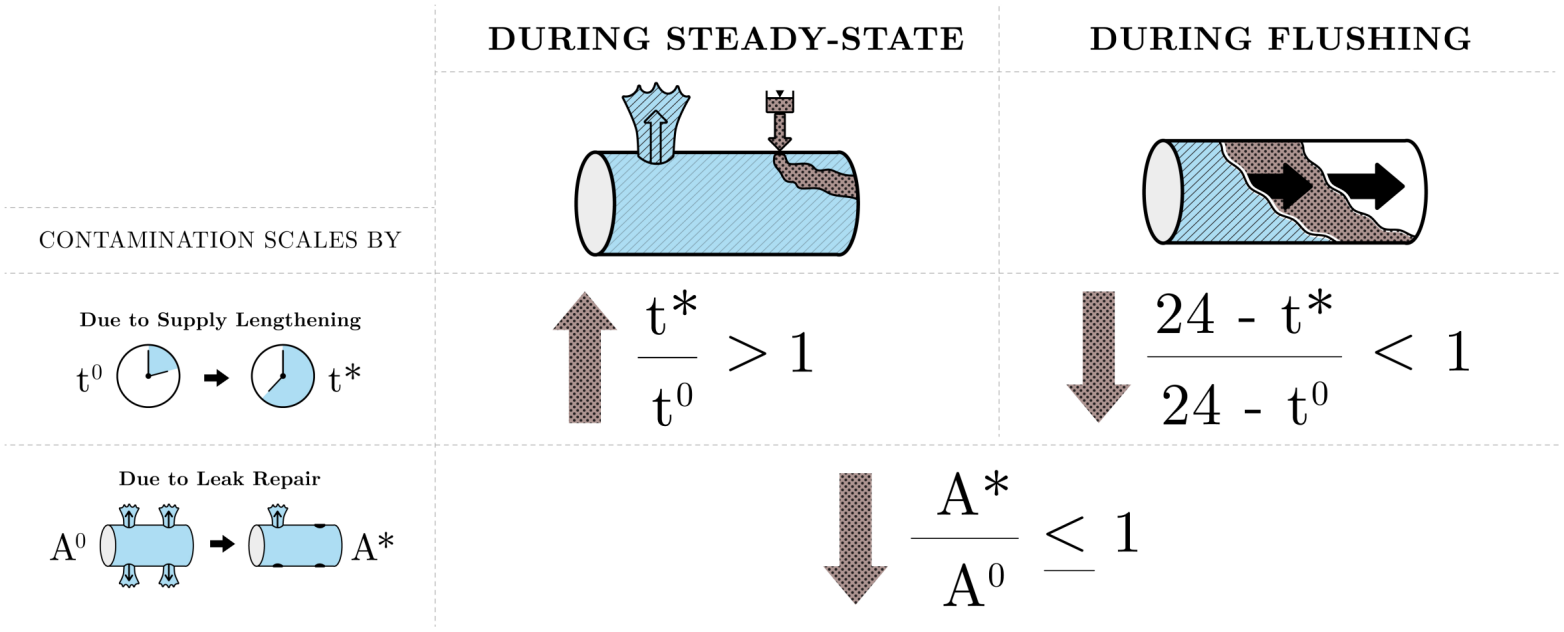
Key scaling relationships governing the risk of intrusion.

The proposed effects of EOA reduction, increased supply duration, and increased supply pressure are summarized in Table 3 and graphically summarized in Fig 4.

**Table 3:** Summary of the scaling equations describing the risk of intrusion. The intruded volume is predicted to scale by the ratio listed in the table when the system’s: supply duration (*t*), pressure head (*H*), or EOA (*A*) is changed from a baseline (^0^) to an improved state (*). Each column assumes that some intruded volume is present in the baseline scenario (i.e., $V_{C}^{0},V_{CF}^{0}>0$).

Due to:	Steady-State $\frac{V_{C}^{*}}{V_{C}^{0}}=$	Flushing Phase $\frac{V_{CF}^{*}}{V_{CF}^{0}}=$	Combined Effect $\frac{V_{C}^{*}+V_{CF}^{*}}{V_{C}^{0}+V_{CF}^{0}}=$
Reduced EOA	$\begin{array}{c} \frac{A^{*}}{A^{0}}\leq \text{min}\left[1,\frac{t^{0}}{t^{*}}{\left(\frac{H^{0}}{H^{*}}\right)}^{\alpha }\left(\frac{l}{pN}+1\right)\right] \end{array}$		
Increased supply duration |A* = *A*^0^, H* = *H*^0^	$\frac{t^{*}}{t^{0}}>1$	$\frac{24-t^{*}}{24-t^{0}}<1$	$\frac{t^{*}(\frac{Q_{C}}{Q_{CF}}-1)+24}{t^{0}(\frac{Q_{C}}{Q_{CF}}-1)+24}\underset{\left(\text{if}\;Q_{CF}\geq Q_{C}\right)}{\leq 1}$
Increased supply pressure |A* = *A*^0^, t* = *t*^0^	$\frac{\phi (H_{C}-H^{*})}{\phi (H_{C}-H^{0})}\leq 1$	None (i.e., 1)	Eq S7 in S2 Text

## Quantitative implications for global IWS

Having defined a set of equations that relate supply duration and pressure, EOA, and intruded volumes, we now consider their implications using benchmarking data from IBNET and BDBWUI and project targets typical of India.

Official Indian targets for city water pipe networks are 17m of pressure and 24 hrs/day of supply duration [39]. We relax the supply duration target to 23.75 hrs/day to: i) distinguish flushing vs. steady-state phases; and ii) enable definition of LR (if 100% of the intruded volume is removed from the flushing phase, *LR* = ∞). Attaining 23.75 hrs/day of supply would still be a major accomplishment for most IWS.

Neither dataset reports the fraction of NRW that is due to physical leakage (i.e., *p*), nor the allowable increases in leakage (i.e., *l*). Since the proposed equations depend on the ratio of $\frac{l}{p}$, we simulate utilities under two scenarios: i) where physical losses are a small percentage of NRW (*p* = 1/3) such that leakage can be allowed to increase substantially (*l* = 0.1; $\frac{l}{p}=0.3$); and ii) where physical losses are 50% of NRW (*p* = 0.5) and the increase in leakage is constrained (*l* = 0.01; $\frac{l}{p}=0.02$). The second scenario is more typical of conditions in South Asia [40].

### Required EOA reductions

**Fig 5:**
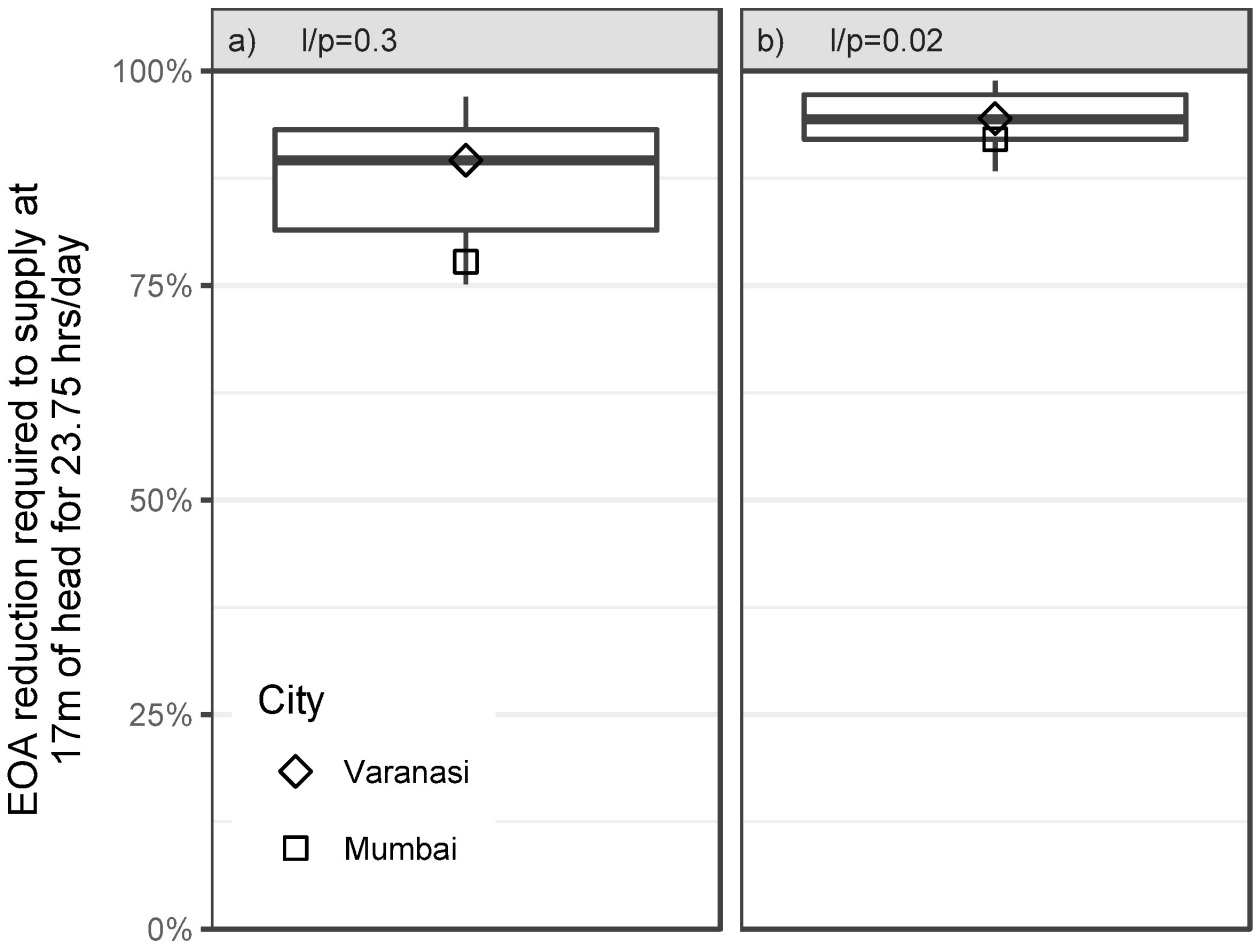
Required EOA reductions in BDBWUI. Our model suggests that increasing the supply duration to 23.75 hrs/day and the pressure to 17m will require utilities to reduce their EOA by a fraction (y-axis) that depends on how much of their current NRW is physical loss (*p*) and their allowed leakage increases (*l*). Box plots summarize these required EOA reductions under scenarios i) and ii) (i.e., a) $\frac{l}{p}=0.3$ and b) $\frac{l}{p}=0.02$).

As supply duration and pressure increase, the nine Indian utilities in our filtered BDBWUI database (Table 1) will have to reduce their EOA by varying amounts; our model’s predictions are summarized in Fig 5. Our model predicts that in scenario i), the majority of Indian utilities will require more than 90% reduction in EOA in order to achieve their pressure and supply duration targets (Fig 5). With more reasonable assumptions in scenario ii), the required reduction in EOA predicted by our model increases to a median of 94%. Varanasi was the median city for these calculations (shown as a sample calculation in S3 Text.). Mumbai reported a lower initial NRW and a higher initial pressure than Varanasi; this lowered our prediction of its required reduction in EOA from 90% to 78% in scenario i) and from 94% to 92% in scenario ii) (shown in Fig 5).

**Fig 6:**
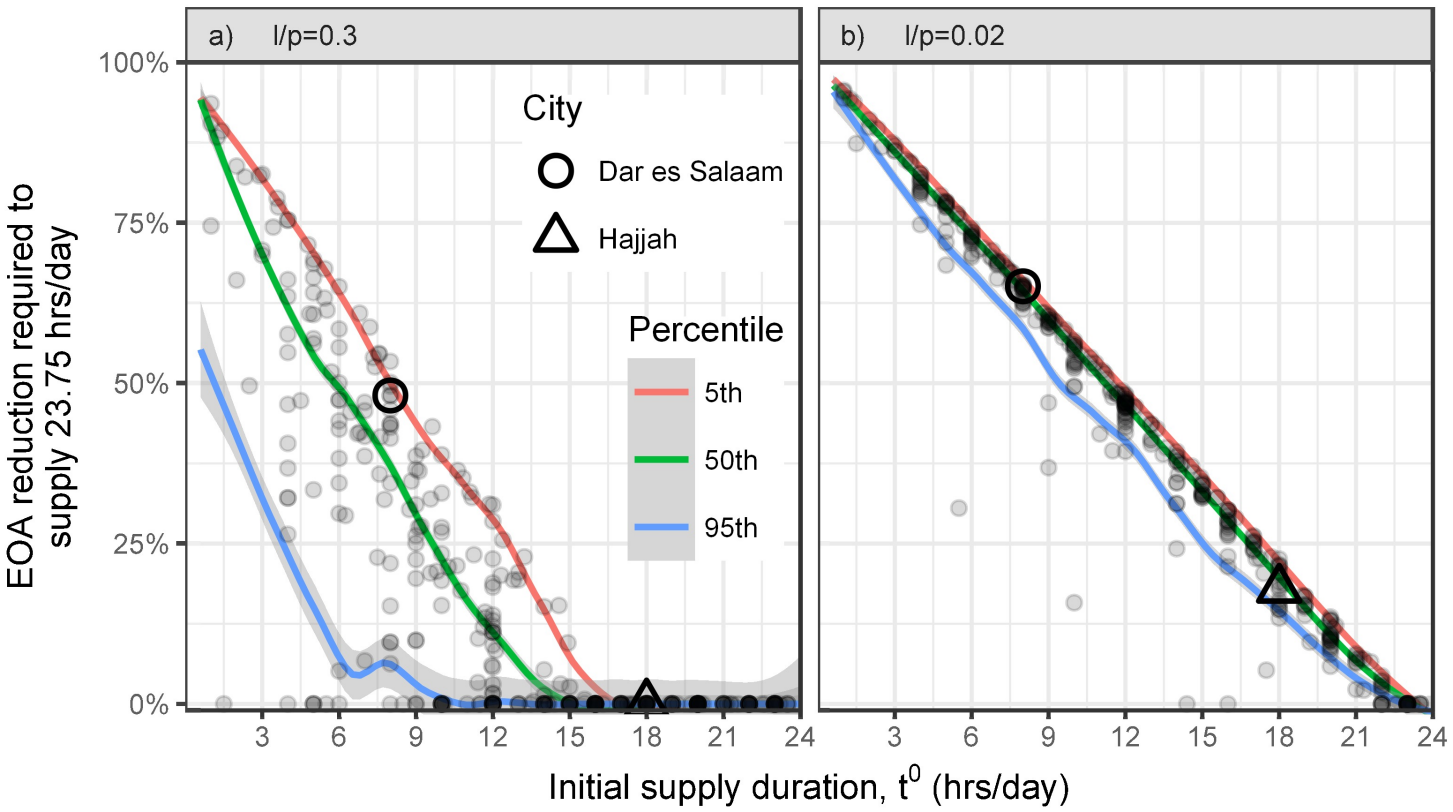
Required EOA reductions in IBNET. Our model predicts that increasing the supply duration to 23.75 hrs/day will require utilities (grey dots) to reduce their EOA by a percentage (y-axis) that depends on their initial supply duration (x-axis) and the ratio of allowed leakage increases (*l*) to the percent of their current NRW that is leakage (*p*). We plot each utility under scenarios i) and ii) (i.e., a) $\frac{l}{p}=0.3$ and b) $\frac{l}{p}=0.02$). For initial supply durations within a 1-hour span (e.g., 4.5-5.5 hrs/day), the 5th (red/upper line), 50th (i.e., median, green/middle line), and 95th (blue/lower line) percentiles are smoothed into the displayed curves.

The IBNET database does not include data on supply pressures so we predict and plot only the EOA reductions necessitated by increasing the supply period duration (Fig 6). To assist the reader in aggregating the many data points, we display a moving average of the 5th, 50th, and 95th percentile of utilities with initial supply durations in a one-hour window. For example, in Fig 6a, utilities with initial supply durations between 2.5 and 3.5 hrs/day are predicted to require a median EOA reduction of 70%, with the 5th and 95th percentile being 82% and 32%.

Under scenario i) there is significant spread in the data. The required reduction in EOA for utilities with initial supply durations *t*^0^ ∈ [5, 6] hrs/day ranged from 11-67% for the 5th and 95th percentile of utilities (Fig 6). However, under the more reasonable assumptions of scenario ii), our model shows the required EOA reduction ranged only from 69-77% for the same range of *t*^0^ (Fig 6).

Where additional leakage can be allowed (e.g., because of a recent expansion in production capacity), utilities with relatively high initial supply durations (e.g., Hajjah) will not need to reduce EOA. Conversely, utilities such as Dar es Salaam, with lower initial supply durations and higher NRW are predicted to require almost 50% reductions in EOA due to increased supply durations even under scenario i).

To cut capital costs, government agencies frequently advocate for repairing instead of replacing existing pipes. Our model suggests that under initially short supply durations and low pressures, repairing existing pipes can create benefits. However, when networks transition to high-pressure CWS, our model predicts that extensive reductions in EOA will be required. Therefore, we should expect that replacing most (if not all) pipes will be the economical solution if high-pressure CWS are to be achieved. In India, where our model predicts that EOA must be reduced by a median of at least 90%, full pipe replacement should be the rule, not the exception.

### Effects of increased supply duration and EOA reduction

**Fig 7:**
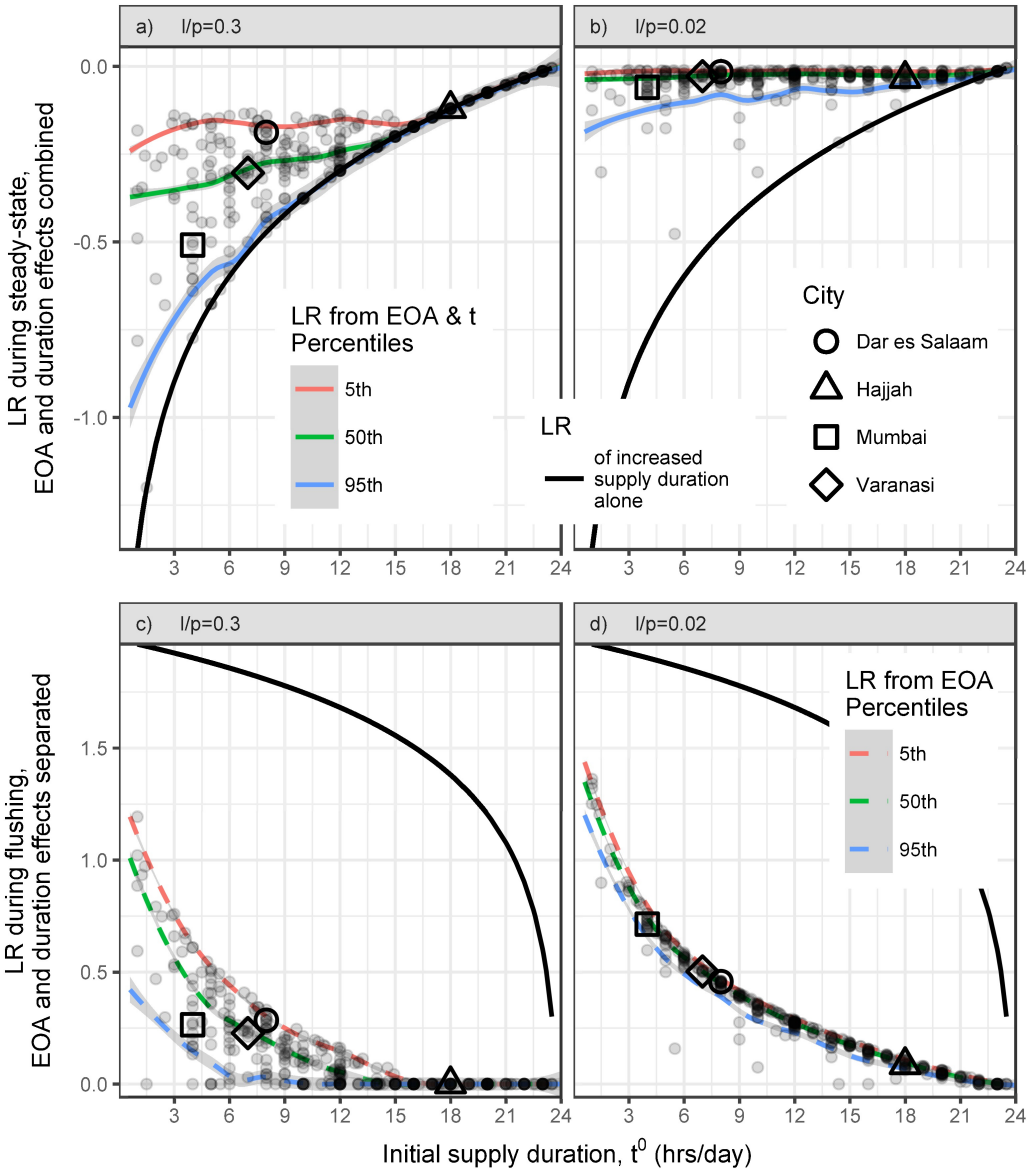
Which improvements reduce the risk of intrusion? For each utility (grey dot) in our filtered IBNET and BWBWUI database, we plot the predicted log reduction (LR) of intruded volume in steady-state (a & b) and flushing (c & d) phases, attributable to increasing the supply duration to 23.75 hrs/day (thick black line) and attributable to the necessitated EOA reductions. For steady-state (a & b), the effects are considered together (solid thin lines), but for the flushing phase (c & d), the effect of EOA reduction is shown separately (dashed thin lines). We plot each utility under scenarios i) and ii) (i.e., $\frac{l}{p}=0.3$ (a & c) and $\frac{l}{p}=0.02$ (b & d)). For initial supply durations within a one-hour span (e.g., 4.5-5.5 hrs/day), the 5th (red/upper line), 50th (i.e., median, green/middle line), and 95th (blue/lower line) percentiles are smoothed into the displayed curves.

Fig 7a depicts the effects predicted by Eq 14 for each utility in our combined IBNET and BSBWUI database. For utilities that allow leakage to increase substantially (scenario i), Eq 14 suggests that increasing the supply duration increases the intrusion risk (i.e., the intruded volume) in the steady-state phase. For example, our model predicts that upgrading the system in Dar es Salaam would cause an increase in the intrusion-induced risk during the steady-state phase. Specifically, it predicts LR = −0.47 from increased supply, LR = +0.28 from required EOA reduction for a net of LR = −0.19 (see S3 Text for a worked example of this calculation). The model predicts that no reduction in EOA is required in Hajjah, and LR = −0.12 is the same as that due to increased supply duration alone. Mumbai had the lowest (i.e., most negative) predicted LR during steady state among our four case study cities; due to its low initial NRW, it requires less EOA reduction.

When leakage cannot increase substantially (scenario ii), the predicted benefits from EOA reduction eliminate most of the predicted harm of increased supply duration. Fig 7b shows the combined values of LR = −0.01 and −0.03 for Dar es Salaam and Hajjah, respectively, during steady-state operations.

The difference between the two scenarios is evident if we consider, for example, utilities with 11.5-12.5 hrs/day of initial supply. Under scenario i), converting to CWS is predicted to increase the intruded volume present during steady-state by a median LR = −0.24 (90% confidence interval: (−0.15, −0.30); Fig 7a). However, under scenario ii), the predicted LR could be held to a median of −0.02 (−0.01, −0.07) (Fig 7b).

Increased supply duration is predicted to reduce the intruded volume present in the flushing phase by LR = 1-2 for all utilities with *t*^0^ ≤ 21 (Fig 7c). Even when utilities cannot allow leakage to substantially increase (scenario ii), increased supply duration is predicted to reduce the intruded volume present in the flushing by more than one order of magnitude more than the reduction in EOA it necessitated (i.e., Δ*LR* ≥ 1) for utilities with *t*^0^ ≥ 2 hrs/day (Fig 7d).

This numerical application of the proposed model reinforces its key prediction: extensive EOA reduction is required to preserve steady-state water safety. It also shows that increased supply durations are an appropriate focus for projects that wish to focus specifically on improving the safety of the flushing water, perhaps to benefit disadvantaged customers in the network. In order to measure the efficacy of such projects, however, utilities should sample during the flushing phase.

### Effects of increased pressure

**Fig 8:**
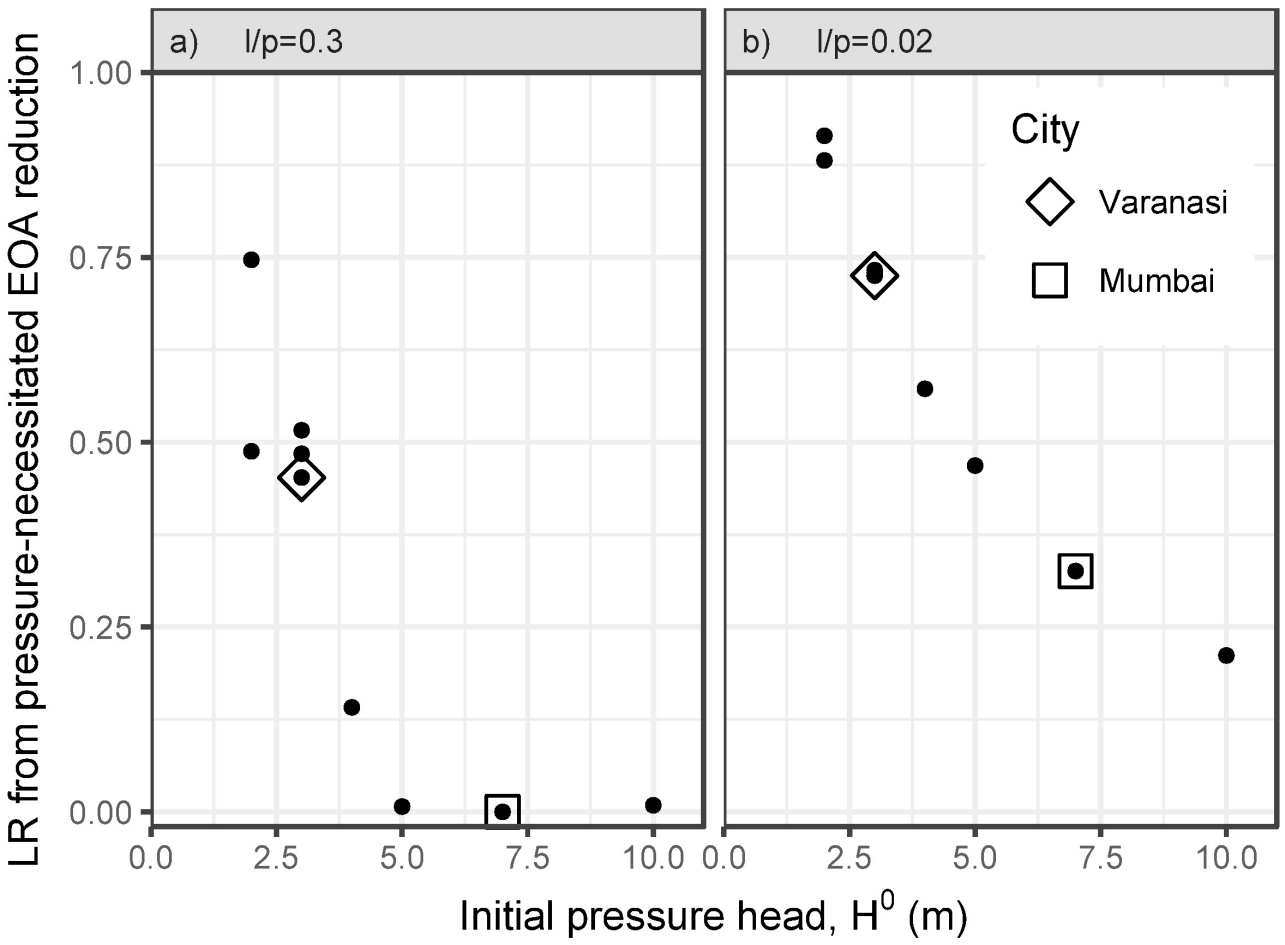
LR from pressure-necessitated EOA reduction. As utilities (dots) in the filtered BDBWUI database increase supply pressures from their initial pressures (x-axis) to 17m, the proposed model suggests that EOA must be reduced. Different EOA reductions are required under scenarios i) and ii) (i.e., a) $\frac{l}{p}=0.3$, and b) $\frac{l}{p}=0.02$). These reductions in EOA are predicted to translate to LR in the intruded volume (y-axis).

Due to the unknown form of *ϕ*() (which stems from the unknown form of *f*() in Eq 3), we limit our analysis to the LR induced by the pressure-necessitated reductions in EOA. These are predicted to be equal in the steady-state and flushing phases (Eq 11). For the two scenarios, we plot the predicted LR for utilities in the BDBWUI due to the decrease in EOA required for H* = 17*m* as a function of initial supply pressure *H*^0^ (Fig 8).

Under scenario i), our model predicts that Mumbai does not require any EOA reduction to transition from its current supply pressure *H*^0^ = 7m to H* = 17m, which implies LR = 0. This prediction is due primarily to Mumbai’s low self-reported NRW. Conversely, Varanasi will require substantial reductions in EOA (LR = 0.45). More generally, under scenario i), cities with *H*^0^ ≤ 3*m* are predicted to have LR ≥ 0.45 due to the EOA reductions necessitated by increasing the pressure (Fig 8a). For scenario ii), this effect is predicted to increase to a LR ≥ 0.72 (Fig 8b).

More generally, our model predicts that utilities that plan to increase their supply pressure to meet targets will need to undergo leak repair campaigns, unless they have very low current NRW levels and can allow leakage to increase. Where increased pressure is proposed to reduce intrusion, our model suggests that leak repair should be undertaken first and then if necessary supplemented with pressure increases.

## Conclusions and recommendations

The proposed and applied scaling equations, summarized in Table 3 and Fig 4, provide insights about how utilities may be able to improve their system performance and reduce their intrusion risk. This paper uses the proposed model to suggest previously-unexplored couplings between system variables. The model suggests that utilities, regulators, and academics alike should take care in distinguishing between water quality during steady-state and flushing phases, and in specifying performance metrics that do not conflict with one another. Hence we highlight six key implications and six opportunities for future work:

**Key implications**
1. Distinguishing between IWS’ predicted effects on flushing and steady-state quality is not something reported in the literature to date and may help understand the health benefits of CWS.
2. Sampling requirements should specify which phase of supply is to be tested. By default, utilities sample steady-state water quality, which typically is of higher quality. However, flushing phase water is often used by disadvantaged customers; its quality cannot be neglected.
3. For utilities in India that are currently planning to convert to high-pressure CWS, our model suggests that full pipe replacement should be the rule, not the exception.
4. Increasing supply duration is likely important for projects focused on improving flushing water quality. However, to preserve water quality during the steady-state phase, our model suggests that such projects should also undergo substantial reductions in EOA.
5. Where increased pressure is proposed to reduce intrusion, our model suggests that EOA reduction should be done first and then, if necessary, supplemented by pressure increases.
6. Targets of increased supply duration, increased pressure, and decreased NRW conflict. The EOA metric eliminates this conflict and better indicates pipe quality.

**Future work**
1. This paper presents the potential implications of our proposed model; testing the model and its predictions in field experiments would validate it and could significantly influence IWS improvement policies and practices.
2. Quantifying the relative contributions of contaminant intrusion, contaminant regrowth, and biofilm sloughing to steady-state water contamination would help identify the key priorities for achieving safer IWS.
3. Investigating which customers consume flushing water (and why) could lead to innovative methods of reducing IWS’ health burden.
4. Determining the relationship between intruded volume and average system pressure would allow this model to be substantially extended.
5. Extending the model developed in this paper to include the coupling of supply duration and pressure should be the next refinement of the proposed equations. This could be implemented by simulating friction, leakage, and contaminants distributed along the length of a single pipe.
6. Water quality degradation is only one way in which IWS negatively affects households [41]. Supplementing this model with additional impacts of IWS would give a more holistic picture of the effects of IWS.

## Notation

For the reader’s convenience, Table 4 summarizes the notation used in this paper.

**Table 4:** Symbols used in this paper, their units, and descriptions.

Symbol	Units	Description
*A*	*m*^2^	Equivalent (cross-sectional) area of an orifice whose leakage rate equals the system’s
*f*()	*m* → −	The (unknown) probability distribution of pressure head within a network
*f*_*C*_	−	Fraction of intrusion pathways with external fluids in their vicinity
*H*	*m*	Average system pressure head
*H*_*C*_	*m*	Average pressure head of external fluids at leakage pathways
*k*_*C*_	*m*^1−*β*^/hr	Scaling coefficient in the intrusion equation
*k*_*L*_	*m*^1−*α*^/hr	Scaling coefficient in the leakage equation
*l*	−	Allowable increased in leakage, as a fraction of the supply volume
*N*	−	Fraction of the input supply that is non-revenue water
*p*	−	Fraction of NRW that is physical leakage
*Q*_*d*_	*m*^3^/hr	Total flow rate demanded by customers
*Q*_*C*_	*m*^3^/hr	Flow rate of intruding fluids into the system during the supply period
*Q*_CF_	*m*^3^/hr	Flow rate of intruding fluids into the system during the non-supply period
*Q*_*L*_	*m*^3^/hr	Flow rate of leaks out of the system
*t*	hr	Supply (period) duration
*V*_*d*_	*m*^3^	Total volume demanded by customers
*V*_*C*_	*m*^3^	Volume of intruded fluids (e.g., rain water or sewage) in the system during steady-state
*V*_*CF*_	*m*^3^	Volume of intruded fluids in the system during flushing
*V*_*L*_	*m*^3^	Volume of leaked water during steady-state
*V*_*T*_	*m*^3^	System input volume
*α*	−	Exponent relating internal pressure head to leakage rate
*β*	−	Exponent relating the inward pressure gradient to intrusion rate
*ϕ*()	*m* → *m*/hr	Function relating the probability distribution of internal (pipe) pressures and the average external pressure to the intrusion rate

## Supporting information

S1 Text Additional modeling details. (PDF)

S2 Text Derivations of Eqs S6 and S7. (PDF)

S3 Text Sample calculations. (PDF)

S1 FigPredicted LR during steady-state from increased supply duration and reduced EOA, combined.The increase (negative LR) in the intruded volume in steady-state due to increased supply duration alone (thick black line and square) and combined with the reductions in EOA required by increased supply duration (colored thin lines). Simulated utilities with three levels of allowed leakage increases: no allowed leakage (pink/upper lines), additional leakage equal to 50% of physical losses (i.e., $\frac{l}{pN}=0.5$) (green/middle lines, and triangle), and additional leakage equal to 100% of physical losses (blue/lower lines). Final durations of 15, 21, and 23.75 hrs/day are shown in the top, middle, and bottom panel, respectively. The text’s example utility is also shown (triangle and square).(TIF)
