# Supplementary material for: Analytical scaling relations to evaluate leakage and intrusion in intermittent water supply systems
Source: PLoS One. 2018 May 18;13(5):e0196887. doi: 10.1371/journal.pone.0196887 (PMC5959068; doi:10.1371/journal.pone.0196887)
Supplement: S1 Text — (PDF) [file pone.0196887.s001.pdf]

S1 Text for: “Analytical scaling relations to evaluate leakage and intrusion in intermittent water supply systems”

Additional modeling details

Leaks and intrusion

Leaks: The standard model for leakage rate (Q_L) out of a pipe is the orifice equation [1, 2]:

$$Q_L = C_d A[2g(H - H_C)]^\alpha \tag{S1}$$

where α accounts for the pressure dependency of the flow rate, C_d is a constant that accounts for the shape of the orifice (and corrects units when $\alpha \neq 0.5$), A is the area of the orifice, g is gravitational acceleration, and H and H_C are system (internal) and contaminant (external) pressure heads. A single, rigid, round orifice would have $\alpha = 0.5$, but cracks in pipe networks can open wider with higher pressures, which can be modeled by higher values of α [2]. Additionally, where multiple leaks are aggregated into an equivalent orifice area (EOA) (of size A), a higher value of α can account for elevation differences between the aggregated orifices [3]; α is typically modeled as between 0.5 to 2.5; we follow standard practice and assume $\alpha = 1$ [2]. Further, we construct ratios such that C_d cancels.

During the supply period, the majority of leaks occur in locations without pressurized intrusion sources, and so we neglect the effect of external fluid pressure head on the outward leakage rate ($H_C \approx 0$). By combining constants ($k_L = C_d(2g)^\alpha 3600\text{s/hr}$), holding the system pressure constant, and integrating with respect to the supply duration (t, in hours), the volume of water lost to leakage from a network simplifies to:

$$V_L = t k_L A H^\alpha \tag{S2}$$

We also note that higher pressures are known to increase the rate of pipe bursts and therefore induce a permanent change in A [4]. The orifice equation model for leakage does not account such coupling, and so we highlight this as an opportunity for refinement of future models.

Intrusion: Where external fluids are in the vicinity of a pathway into the pipe, it is common practice to model their rate of intrusion (Q_C) as flow through an orifice [5]:

$$Q_C = C_d A_C [2g(H_C - H)]^{\beta} \tag{S3}$$

where, β accounts for the pressure dependency of intrusion, C_d is an orifice coefficient accounting for the shape of the orifice (and correcting units when $\beta \neq 0.5$), A_C is the size of the orifice where intrusion is occurring, and g, H, and H_C are as in Equation S1.

Since external fluid pressure is not likely to be high enough to act to close the orifice, a single intrusion location likely has $\beta \approx 0.5$ (although this common assumption has not been experimentally investigated [5]). However, if multiple intrusion orifices are aggregated into an equivalent orifice, a different value of beta could account for changes in H between sites.

External fluid pressure head: In CWS, pipes laid below the water table are a common location for intrusion; H_C is often modeled as the pipe depth below the water table [5]. For simplicity however, commercial models for intrusion risk often combine the various pipe depths into a single external fluid pressure head (H_C) [5].

In IWS, many sources of contamination have very low or no pressure associated with them (e.g. moist soils); some sources may have pressure up to the buried depth of the water pipe (e.g. passing through an open sewer or pond); and, occasionally sewers become blocked and can create a pressurized source of raw sewage, which poses a severe intrusion hazard. Similarly, some sections of an IWS have high pressure, while others may have low, or even negative pressure.

Intrusion reduction metrics: log reduction

While some practitioners may be used to measuring reduction in contaminants as a percentage, many chemical and physical disinfection processes are measured by log reduction (LR) [6]. LR accounts for the fact that it is harder to remove the last traces of contamination [6]. Accordingly, we use log reduction (LR) to account for the reduction in the intruded volume from an original volume, V_C^0, to the final volume, V_C^*:

$$\text{LR} = \log_{10}(\frac{V_C^0}{V_C^*}) = -\log_{10}(\frac{V_C^*}{V_C^0}) \tag{S4}$$

LR = 1.0, 2.0, 3.0 correspond to 90%, 99%, and 99.9% reductions in intruded fluids, respectively. LR has the added advantages of making the superposition of multiple effects linear, and increasing the ease of displaying different reduction values. A negative value of LR suggests that the intruded volume has increased.

Dataset details

IBNET: The World Bank's IBNET database holds self-reported performance indicators for 3085 different utilities delivering water to more than 996 million people [7]. Of these utilities, 659 (21%) have supply durations for less than 23.75 hrs/day. These IWS serve a population of 303 million people. Unfortunately, not all of these utilities report NRW, population, and supply duration. We therefore consider only the most recent data from the subset of IWS utilities who report all three.

Following a previous study of the IBNET database [8], we exclude outliers by considering only IWS utilities reporting populations and NRW between the 1st and 99th percentile values. Similarly we exclude IWS reporting supply durations less than the 1st percentile of IWS supply duration. The resultant data set includes 325 utilities (Table 1). Almost half (46%) of the filtered database entries are from Sub-Saharan Africa, and the dataset includes entries from only one South Asian country (Bangladesh). The mean supply duration was 13 hrs/day, and the mean NRW was 36%. Dataset entries occurred from 2001 to 2015 (Table 1). Unfortunately, the IBNET database does not include a metric for (system or customer) pressure.

BDBWUI: We, therefore, supplement IBNET data with self-reported benchmarking data for 20 utilities in India serving 55 million people [9], reported in the 2007 Benchmarking and Data Book of Water Utilities in India (BDBWUI). Helpfully, the BDBWUI data includes a metric for average pressure head at customer connections, measured in meters of water column. The BDBWUI dataset reports leakage in terms of UFW instead of NRW, but as discussed in Section 2.8 we neglect this difference and list it as NRW.

To account for outliers we removed the minimum and maximum values for NRW, pressure, and supply duration, and omitted missing data; the resultant dataset included results from nine utilities serving a total of 13 million people (Table 2).

References

[1] Colombo AF, Karney BW. Energy and costs of leaky pipes: toward comprehensive picture. Journal of Water Resources Planning and Management. 2002;128(6):441–450. doi:10.1061/(ASCE)0733-9496(2002)128:6(441).

[2] American Water Works Association. Water audits and loss control programs. 3rd ed. No. M36 in AWWA manual. Denver, Colorado: American Water Works Association; 2009.

[3] Gupta R, Bhave PR. Comparison of methods for predicting deficient-network performance. Journal of Water Resources Planning and Management. 1996;122(3):214–217.

[4] Thornton J, Lambert A. Progress in practical prediction of pressure: leakage, pressure: burst frequency and pressure: consumption relationships. In: Pro-

ceedings of IWA Special Conference 'Leakage 2005'. Halifax, Canada; 2005. p. 1–11. Available from: http://env1.kangwon.ac.kr/leakage/2009/management/research/knowledge/09/na/Progress%20in%20practical%20prediction%20of%20pressure%20relationships.pdf.

[5] Besner MC, Prévost M, Regli S. Assessing the public health risk of microbial intrusion events in distribution systems: conceptual model, available data, and challenges. Water Research. 2011;45(3):961–979. doi:10.1016/j.watres.2010.10.035.

[6] Benjamin MM, Lawler DF. Water quality engineering: physical/chemical treatment processes. Hoboken, N.J: John Wiley & Sons; 2013.

[7] van den Berg C, Danilenko A. The IBNET water supply and sanitation performance blue book. World Bank; 2011. Available from: dx.doi.org/10.1596/978-0-8213-8582-1.

[8] van den Berg C. Drivers of non-revenue water: a cross-national analysis. Utilities Policy. 2015;36:71–78.

[9] Asian Development Bank, Ministry of Urban Development Government of India. Benchmarking and data book of water utilities in India. Manila: Asian Development Bank; 2007. Available from: http://hdl.handle.net/11540/222.
