# Supplementary material for: Analytical scaling relations to evaluate leakage and intrusion in intermittent water supply systems
Source: PLoS One. 2018 May 18;13(5):e0196887. doi: 10.1371/journal.pone.0196887 (PMC5959068; doi:10.1371/journal.pone.0196887)
Supplement: S2 Text — (PDF) [file pone.0196887.s002.pdf]

S2 Text for: “Analytical scaling relations to evaluate leakage and intrusion in intermittent water supply systems”

Derivation of the effect of reducing the EOA on intrusion

The EOA for leaks (A) is common to Eqs 1, 2, and 4 . Reducing A (and therefore V_L) by the fraction $\frac{A^*}{A^0}$ (in the case where $t^* = t^0$ and $H^* = H^0$), will therefore also scale the volume of intruded fluids in the steady-state and flushing phases by the ratio $\frac{A^*}{A^0}$:

$$
\begin{aligned}
\left.\frac{V_L^*}{V_L^0}\right|_{t^*=t^0, H^*=H^0} &= \frac{t^0 A^* \left(H^0\right)^\alpha}{t^0 A^0 \left(H^0\right)^\alpha} &&= \frac{A^*}{A^0} && : V_L^0 > 0 \\
\left.\frac{V_C^*}{V_C^0}\right|_{t^*=t^0, H^*=H^0} &= \frac{t^0 f_C A^* \phi(H_C - H^0)}{t^0 f_C A^0 \phi(H_C - H^0)} &&= \frac{A^*}{A^0} && : V_C^0 > 0 \\
\left.\frac{V_{CF}^*}{V_{CF}^0}\right|_{t^*=t^0} &= \frac{(24 - t^0) k_C f_C A^* H_C^\beta}{(24 - t^0) k_C f_C A^0 H_C^\beta} &&= \frac{A^*}{A^0} && : V_{CF}^0 > 0 \\
\therefore \left.\frac{V_C^*}{V_C^0}\right|_{t^*=t^0, H^*=H^0} &= \left.\frac{V_{CF}^*}{V_{CF}^0}\right|_{t^*=t^0} = \left.\frac{V_L^*}{V_L^0}\right|_{t^*=t^0, H^*=H^0} &&= \frac{A^*}{A^0} && : V_C^0, V_{CF}^0, V_L^0 > 0
\end{aligned}
\tag{S6}
$$

Conditions for CWS to reduce customers' exposure to intruded fluid

From Eq 13, assuming $t^* > t^0$:

$$\begin{aligned}
&\left.\frac{V_C^* + V_{CF}^*}{V_C^0 + V_{CF}^0}\right|_{A^*=A^0, H^*=H^0} \leq 1 \\
\iff & \frac{t^*\left(\frac{Q_C}{Q_{CF}} - 1\right) + 24}{t^0\left(\frac{Q_C}{Q_{CF}} - 1\right) + 24} \leq 1 \\
\iff & Q_{CF} \geq Q_C \\
\iff & k_C H_C^\beta \geq \phi(H_C - H^0) \\
\iff & k_C H_C^\beta \geq \phi(H_C - H^0) \\
\iff & \int_{-\infty}^{H_C} f(H)\left(1 - \frac{H}{H_C}\right)^\beta dH \leq 1
\end{aligned} \tag{S7}$$

Deriving a simpler sufficient, but not necessary condition, if $H \geq 0$ everywhere, then:

$$\int_{-\infty}^{0} f(H)g()dH \equiv 0 \qquad : \text{any function } g()$$

$$\therefore \int_{-\infty}^{H_C} f(H)\left(1 - \frac{H}{H_C}\right)^\beta dH = \int_{0}^{H_C} f(H)\left(1 - \frac{H}{H_C}\right)^\beta dH \leq 1 \qquad : \beta > 0 \tag{S8}$$

Effect of pressure on steady-state plus flushing phase

For the completeness of the Table 4, we calculate the combined effect of pressure changes on the volume of contaminants in the steady-state and flushing phases combined. It depends on the ratio of the intruded volume in steady-state phase to that in the flushing phase (Eq S9). Independent of the ratio, however, increasing pressure cannot increase the total intruded volume in the system.

$$\frac{V_{C+CF}^*}{V_{C+CF}^0} = \frac{\phi(H_C - H^*) + (\frac{24}{t} - 1)\phi(H_C)}{\phi(H_C - H^0) + (\frac{24}{t} - 1)\phi(H_C)} \tag{S9}$$
