# Supplementary material for: Analytical scaling relations to evaluate leakage and intrusion in intermittent water supply systems
Source: PLoS One. 2018 May 18;13(5):e0196887. doi: 10.1371/journal.pone.0196887 (PMC5959068; doi:10.1371/journal.pone.0196887)
Supplement: S3 Text — (PDF) [file pone.0196887.s003.pdf]

S3 Text for: "Analytical scaling relations to evaluate leakage and intrusion in intermittent water supply systems"

Varanasi

Required reduction in EOA is given by Eq 11. Varanasi reported $N = 0.3$, $H^0 = 3$, & $t^0 = 7$. The target system goals are $t^* = 23.75$ and $H^* = 17$. Therefore, Varanasi's required reduction in EOA is:

$$\frac{A^*}{A^0} = \min\left[1, \frac{t^0}{t^*}\left(\frac{H^0}{H^*}\right)^\alpha \left(\frac{w}{pN}+1\right)\right]$$
$$= \min\left[1, \frac{7}{23.75}\left(\frac{3}{17}\right)^1 \left(\frac{w}{p0.3}+1\right)\right]$$

for Scenario i), $\frac{w}{p} = 0.3$:

$$\therefore \frac{A^*}{A^0} = \min\left[1, \frac{7}{23.75}\left(\frac{3}{17}\right)^1 \left(\frac{0.3}{0.3}+1\right)\right]$$
$$= 0.104 = 90\%\text{decrease} \tag{S8}$$

for Scenario ii), $\frac{w}{p} = 0.02$:

$$\therefore \frac{A^*}{A^0} = \min\left[1, \frac{7}{23.75}\left(\frac{3}{17}\right)^1 \left(\frac{0.02}{0.3}+1\right)\right]$$
$$= 0.055 = 94\%\text{decrease} \tag{S9}$$

Dar es Salaam

LR in the intruded volume in the steady-state phase due to increased supply duration and EOA reduction is given by Eq 15. Dar es Salaam reported $N = 0.56$ and $t^0 = 8$. The target system goal is $t^* = 23.75$. Therefore, Dar es

Salaam's LR during steady state is:

$$LR = -log_{10}(\frac{V_C^*}{V_C^0}\bigg|_{H^*=H^0}) = -log_{10}\left[\frac{t^*}{t^0}\min\left[1, \left(\frac{t^0}{t^*}\right)\left(\frac{w}{pN}+1\right)\right]\right]$$
$$= -log_{10}(\frac{t^*}{t^0}) + \min\left[0, -log10\left[\left(\frac{t^0}{t^*}\right)\left(\frac{w}{pN}+1\right)\right]\right]$$

for Scenario i), $\frac{w}{p} = 0.3$:

$$\therefore LR = -log_{10}(\frac{23.75}{8}) + \min\left[0, -log10\left[\left(\frac{8}{23.75}\right)\left(\frac{0.3}{0.56}+1\right)\right]\right]$$
$$= -0.47 + 0.29 = -0.18 \tag{S10}$$

for Scenario ii), $\frac{w}{p} = 0.02$:

$$\therefore LR = -log_{10}(\frac{23.75}{8}) + \min\left[0, -log10\left[\left(\frac{8}{23.75}\right)\left(\frac{0.02}{0.56}+1\right)\right]\right]$$
$$= -0.47 + 0.46 = -0.01 \tag{S11}$$
