# Supplementary figures and images for: Analytical scaling relations to evaluate leakage and intrusion in intermittent water supply systems
Source: PLoS One. 2018 May 18;13(5):e0196887. doi: 10.1371/journal.pone.0196887 (PMC5959068; doi:10.1371/journal.pone.0196887)

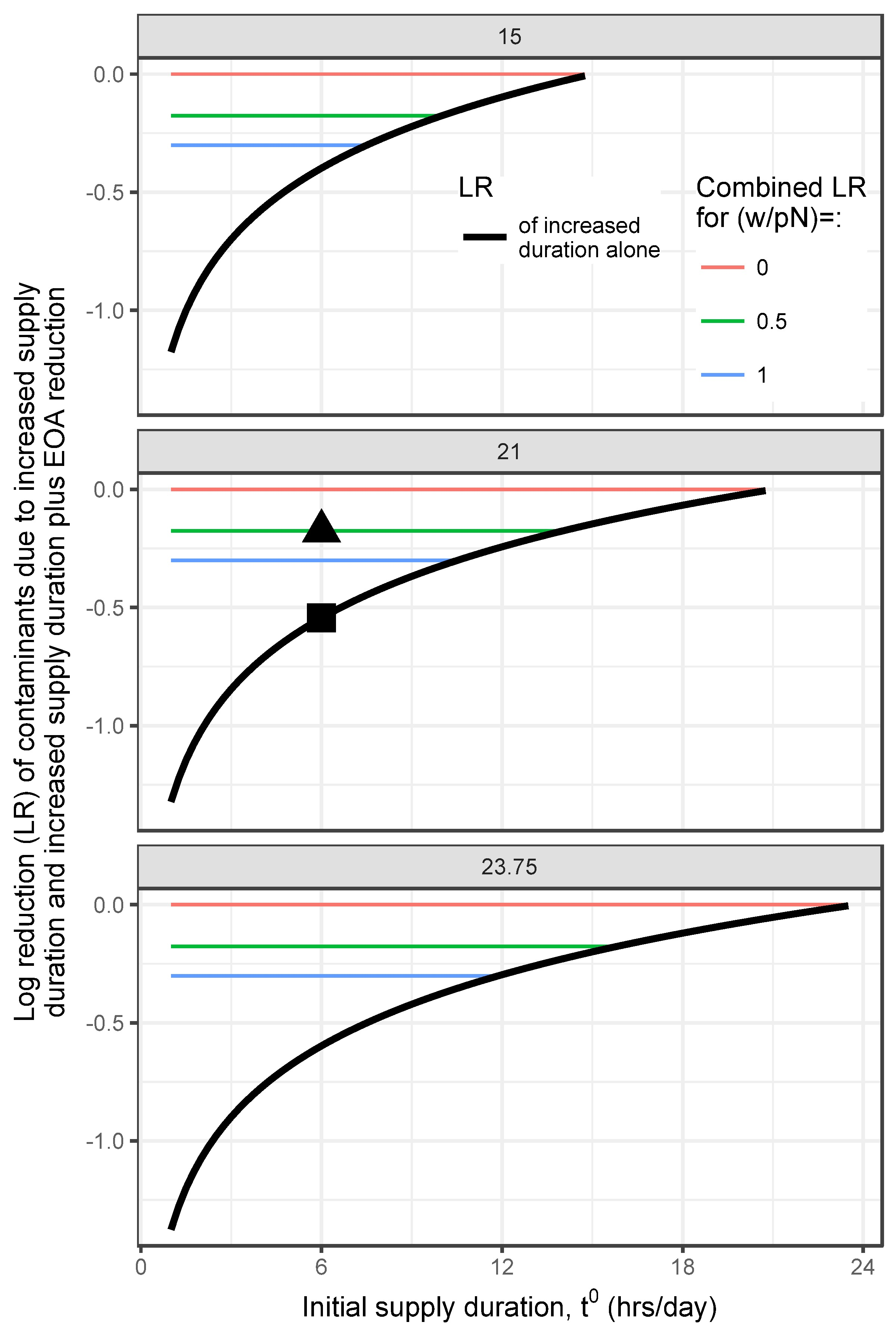

Supplement: S1 Fig — The increase (negative LR) in the intruded volume in steady-state due to increased supply duration alone (thick black line and square) and combined with the reductions in EOA required by increased supply duration (colored thin lines). Simulated utilities with three levels of allowed leakage increases: no allowed leakage (pink/upper lines), additional leakage equal to 50% of physical losses (i.e., lpN=0.5) (green/middle lines, and triangle), and additional leakage equal to 100% of physical losses (blue/lower lines). Final durations of 15, 21, and 23.75 hrs/day are shown in the top, middle, and bottom panel, respectively. The text’s example utility is also shown (triangle and square). (TIF) [file pone.0196887.s004.tif]
